# Chalcone Derivatives From *Abelmoschus manihot* Seeds Restrain NLRP3 Inflammasome Assembly by Inhibiting ASC Oligomerization

**DOI:** 10.3389/fphar.2022.932198

**Published:** 2022-07-07

**Authors:** Jinsong Su, Fujing Yang, Xuemei Kang, Jia Liu, Yiwen Tao, Qingchun Diao, Xianli Meng, Deming Liu, Yi Zhang

**Affiliations:** ^1^ School of Ethnic Medicine, and Research Institute of Integrated TCM and Western Medicine, Chengdu University of Traditional Chinese Medicine, Chengdu, China.; ^2^ Chongqing Clinical Research Center for Dermatology, Chongqing Key Laboratory of Integrative Dermatology Research, Key Laboratory of External Therapies of Traditional Chinese Medicine in Eczema, Department of Dermatology, Chongqing Traditional Chinese Medicine Hospital, Chongqing, China.; ^3^ Hospital Administration Office, The First People’s Hospital of Ziyang, Ziyang, China.

**Keywords:** *Abelmoschus manihot*, chemical composition, chalcone derivative, abelmanihotols A-C, NLRP3 inflammasome

## Abstract

Three chalcone derivatives, abelmanihotols A−C **(1–3)**, and nine known compounds were isolated from *A. manihot* seeds, and their structures were determined using HRESIMS and NMR spectroscopic analysis. Compound **1** exhibited the most potent inhibitory effect (IC_50_ = 4.79 ± 0.72 μM) against lipopolysaccharide (LPS)-induced NO release in THP-1 cells, and **s**ignificantly inhibited interleukin 1β (IL-1β) secretion, which is stimulated by LPS plus nigericin (IC_50_ = 11.86 ± 1.20 μM), ATP or MSU, in THP-1 cells. A preliminary mechanism of action study indicated that compound **1** blocked the formation of nucleotide oligomerization domain-like receptor protein-3 (NLRP3) inflammasome formation by suppressing apoptosis-associated speck-like protein oligomerization, thereby attenuating caspase-1 activation and IL-1β release. These results reveal that compound **1** is not only a potent and efficacious NLRP3 inflammasome inhibitor but also a promising drug for the treatment of NLRP3-related diseases.

## Graphical Abstract

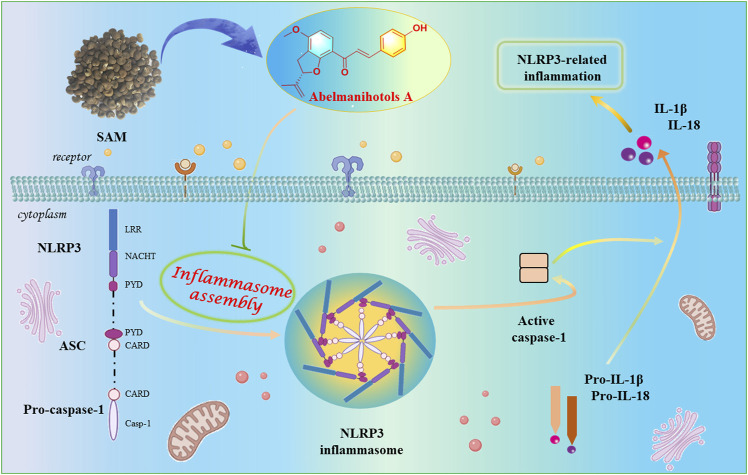

## Introduction

As an important component of innate immunity, the nucleotide oligomerization domain-like receptor protein-3 (NLRP3) inflammasome plays an important role in the body’s immune responses, which are closely related to the occurrence and development of diseases (Shen et al., 2018; Fusco et al., 2020). Aberrant interleukin 1β (IL-1β) secretion and NLRP3 inflammasome formation are closely related to metabolic diseases and inflammatory responses, including obesity, inflammatory bowel diseases, atopic dermatitis, psoriasis, and rheumatoid arthritis. Inflammasome priming and inflammasome assembly are the two sequential signals in NLRP3 inflammasome activation (Wang et al., 2020). In the priming step, immune cells receive priming stimuli, such as the binding of lipopolysaccharides (LPSs) to Toll-like receptors 4 (TLR4), to activate nuclear factor kappa-B (NF-κB) and then up-regulate NLRP3 and pro-IL-1β mRNA expression. After receiving the priming signal, various factors, such as pathogen-associated molecular patterns (PAMPs) or damage-associated molecular patterns (DAMPs), initiate the NLRP3 inflammasome activation (Kim and Jo, 2013). NLRP3 binds to the PYD domain of the apoptosis-associated speck-like protein (ASC), and then the CARD domain of ASC binds to the CARD on pro-caspase-1 to form a complete and activated NLRP3 inflammasome, which promotes the self-cleavage of pro-caspase-1 and produces an active effect protein caspase-1. Caspase-1 cleaves GSDMD, releasing the N-terminal domain of GSDMD, which binds to phosphatidylserine and phosphoinositide on the inner pages of the cell membrane, where it punches holes, causing cell death and the release of cell contents (Sagulenko et al., 2013; Sharif et al., 2019). In addition to cleaving GSDMD, caspase-1 induces the transformation of IL-1β and IL-18 from an immature to an active state. After cell death, IL-1β and IL-18 are released outside the cell to induce inflammation. Hence, NLRP3 inflammasome inhibitors are important for the treatment of related diseases.

The plant *A. manihot* (L.) Medic., belonging to the mallow family (Malvaceae), is a traditional Tibetan medicine and is documented in *Jing Zhu Ben Cao*. The plant *A. manihot* is mainly distributed in valleys, ditches, roadsides, and wilderness grasses at an altitude of 150–1,200 m in China. According to the theory of Tibetan medicine, the seed of *A. manihot* (སོ་མ་ར་ཛ། , SAM) has been used for the treatment of atopic dermatitis, rheumatism, and leprosy, and contains chalcone, which has antibacterial, antifungal, anticancer, antioxidative, and anti-inflammatory effects (Ohnogi et al., 2012). In the present research, three new chalcone derivatives abelmanihotols A−C (compounds **1–3**), and nine known compounds (compounds **4–12**) were isolated from SAM. Herein, we explain their isolation, structure, and the potential mechanism by which such compounds restrain NLRP3 inflammasome activation.

## Materials and Methods

### General Experimental Procedures

Optical rotations were measured with a Horiba SEPA-300 polarimeter (Horiba, Kyoto, Japan). UV spectra were recorded on a Shimadzu UV-2401PC spectrometer (Shimadzu Corporation, Tokyo, Japan). For column chromatography, C-18 silica gel (40 μm × 63 μm; Daiso Co., Japan), MCI gel CHP 20P (75 μm × 150 μm, Mitsubishi Chemical Industries, Tokyo, Japan), and Sephadex LH-20 (Amersham Pharmacia, Uppsala, Sweden) were employed. Semipreparative HPLC was performed using an Agilent Zorbax SB-C18 (250 mm × 9.4 mm, i.d., 5 μm). NMR spectra were recorded on a Bruker Avance III 700 MHz spectrometer (Bruker, Karlsruhe, Germany), or a Bruker Avance III 600 MHz spectrometer, and TMS was used as an internal standard. HREIMS data were collected with an Agilent G6230 TOF MS spectrometer (Agilent Technologies, Santa Clara, CA, United States).

### Plant Material

SAMs were collected from the Hongyuan area of Sichuan Province, China, in August 2019. The plant was identified by Prof. Yi Zhang, School of Ethnic Medicine, Chengdu University of Traditional Chinese Medicine, China. A voucher specimen (No. 20190817001) was deposited at the School of Ethnic Medicine, Chengdu University of Traditional Chinese Medicine, China.

### Extraction and Isolation

Dried and powdered SAMs (10 kg, dry weight) were extracted with an 85% ethanol (80 L × 4 × 24 h) immersion extraction. The crude extract (600 g) was suspended in water (2 L) and partitioned with ethyl acetate (EtOAc) three times for the preparation of an EtOAc extract (100 g), which was further subjected to a chromatographic column (SiO_2_, petroleum ether/EtOAc, 500:1, 40 L, 300:1, 40 L, 100:1, 40 L, 50:1, 40 L, 30:1, 40 L, 10:1, 40 L, 5:1, 40 L, and 1:1, 40 L) to provide three portions (fraction 1-fraction 3, Fr.1–Fr.3).

We separated Fr.1 (8 g) with Sephadex LH-20 (MeOH) to obtain five portions (Fr.1.1–Fr.1.5). Fr. 1.4 (1.2 g) was purified through semipreparative HPLC with a 20% MeOH/H_2_O system (3 ml/min) to afford **7** (3.2 mg, t_R_ = 17.0 min). Fr. 1.5 (2.0 g) was separated with Sephadex LH-20 (MeOH) to produce eight portions (Fr.1.5.1–Fr.1.5.8). We further purified the fraction of Fr.1.5.1 (230 mg) through semipreparative HPLC (25%, MeOH/H_2_O, flow rate: 3 ml/min) to obtain **8** (3.7 mg, t_R_ = 8.0 min). Fr.1.5.2 (240 mg) was subjected to semipreparative HPLC (25%, MeOH/H_2_O, flow rate: 3 ml/min) to produce **1** (4.5 mg, t_R_ = 12.4 min). Fr.1.5.3 (160 mg) was subjected to semipreparative HPLC (25%, MeOH/H_2_O, flow rate: 3 ml/min) to produce **10** (1.3 mg, t_R_ = 26.0 min). Fr.1.5.4 (170 mg) was subjected to semipreparative HPLC (27%, MeOH/H_2_O, flow rate: 3 ml/min) to produce **9** (2.6 mg, t_R_ = 18.4 min). Fr.1.5.5 (260 mg) was subjected to semipreparative HPLC (29%, MeOH/H_2_O, flow rate: 3 ml/min) to produce **2** (4.7 mg, t_R_ = 22.4 min). Similarly, Fr.1.5.8 (350 mg) was subjected to semipreparative HPLC (40%, MeOH/H_2_O, flow rate: 3 ml/min) to produce **11** (1.6 mg, t_R_ = 34.0 min), **12** (1.9 mg, t_R_ = 28.5 min), and **3** (2.4 mg, t_R_ = 38.0 min). Fr.3 (4 g) was separated with Sephadex LH-20 (MeOH) to produce two portions (Fr.3.1 and Fr.3.2). Fr.3.2 (110 mg) was subjected to semipreparative HPLC (28%, MeOH/H_2_O, flow rate: 3 ml/min) to produce **6** (1.3 mg, t_R_ = 24.2 min). Fr.4 (7 g) was separated with Sephadex LH-20 (MeOH) to produce two portions (Fr.4.1 and Fr.4.2). Fr.4.2 (350 mg) was subjected to semipreparative HPLC (25%, MeOH/H_2_O, flow rate: 3 ml/min) to produce **5** (2.3 mg, t_R_ = 16.2 min) and **4** (3.5 mg, t_R_ = 24.0 min).

### Cell Culture and Regents

A human acute monocytic leukemia (THP-1) cell line was obtained from Procell Life Science and Technology (Wuhan, China) and cultured at 37°C and 5% CO_2_ in a RPMI-1640 medium, supplemented with a 10% FBS and 1% penicillin/streptomycin solution. Phorbol 12-myristate 13-acetate (PMA), nigericin, adenosine triphosphate (ATP), lipopolysaccharide (LPS), and monosodium urate (MSU) were purchased from Sigma (Shanghai, China). VX-765 and BAY 11-7085 were purchased from Selleck (Shanghai, China).

### Cell Viability Assay

When THP-1 cell reached 90% confluency in a cell plate, 6×10^3^ THP-1 cells were seeded in each of 96-well plates with 10 nM PMA. After the cells adhered, the medium was replaced with a new medium having different doses of compounds or of DMSO, and incubated for another 24 h. Then, 10% CCK-8 (V/V) was added to the medium, and the cells were incubated for another 1 h. The absorbance was detected with VARIOSKAN LUX (Thremo Scientific, Waltharm, MA, United States) at 450 nm.

### Detection of NO Production

When THP-1 cell reached 90% confluency in a cell culture plate, 6 × 10^3^ THP cells were seeded in 96-well plates with 10 nM PMA. After the cells adhered, the medium was replaced with a new medium containing compounds **1–12** (10 μΜ), BAY 11-7085 (10 μΜ) or DMSO and incubated for 2 h. Then LPS (0.5 μg/ml) was added to each well, except the DMSO group, and the cells were incubated for another 24 h. The supernatant was collected, and NO levels were measured according to the kit manufacturer’s instructions (Solarbio Life Science, Beijing, China).

### Measurement of IL-β Release

When THP-1 cell reached 90% confluence in a cell culture plate, 3 × 10^4^ THP-1 cells were seeded in six-well plates with 100 nM PMA. After the cells differentiated into macrophages, they were stimulated with 1 μg/ml LPS for 3 h. Thereafter, the THP-1 cells were treated with compound **1** (5, 10, and 20 μM) for 30 min, and then stimulated with nigericin (5 μM), MSU (150 mg/ml) for another 6 h, or ATP (5 mM) for 45 min. The supernatants of the cell culture were collected and assayed according to the kit manufacturer’s instructions (Elabscience, Wuhan, China).

### Western Blotting

After corresponding treatment, the THP-1 cells were washed three times with cold PBS and then lysed in a RIPA buffer. The protein concentration was detected using a BCA kit (Beyotime, Shanghai, China). 20 µg denatured protein in each group was separated using an SDS-PAGE and then transferred to PVDF membranes. The membranes were incubated with the corresponding primary antibody overnight, with GAPDH serving as the control.

### ASC Staining Detection

When THP-1 cell reached 90% confluency in a cell culture plate, 3 × 10^5^ THP-1 cells were seeded in each 12-well plate. After compound **1** treatment, the cells were washed three times with PBS, fixed in 4% paraformaldehyde, and permeabilized with 0.1% Triton X-100. Then the cells were incubated with an anti-ASC primary antibody (Proteintech, Wuhan, China), a secondary goat anti-rabbit IgG labeled with Cy3 dye (proteintech), and then stained with DAPI (proteintech). Images were obtained on a Leica TCS SP8 (Leica, Wetzlar, Germany).

### Statistical Analysis

All data were presented as means ± standard deviations of three replicates and calculated with GraphPad Prism 6.0 software (GraphPad, La Jolla, CA, United States). Difference was considered statistically significant at a probability value of <0.05.

## Results

### Structural Elucidation

The separation of the EtOAc extract of SAM led to the isolation of three new chalcone derivatives: abelmanihotols A−C (**1–3**) and nine known compounds: isobavachalcone (**4**) (Lin et al., 2016), xanthoangelol (**5**) (Wang and Zhang, 2018), 4-hydroxyderricin (**6**) (Wang and Zhang, 2018), xanthoangelol D (**7**) (Baba et al., 1990), jejuchalcone E (**8**) (Shimomura et al., 2013), 1-[2,3-Dihydro-2-(1-hydroxy-1-methylethyl)-4-methoxy-7-benzofuranyl]-3-(4-hydroxyphenyl)-2-propen-1-one (**9**) (Ohnogi et al., 2012), 4′-methoxy-bavachromanol (**10**) (Kumazawa et al., 2006), xanthoangelol G (**11**) (Park et al., 2016), and (1-[2-hydroxy-3-(7-hydrox-3,7-dimethyl-2,5-ocadienyl)-4-methoxyphenyl]-3-(4-hydroxyphenyl)-2-propen-l-one chalcone (**12**) (Ryu and Kweon, 2018).

Compound **1**: yellow solid; UV (MeOH) λmax (logε) 365 (4.4) nm; ${\left[\alpha \right]}_{D}^{25.0}\;–$ 25.8 (c 0.03, MeOH); HRESIMS m/z = 337.1437 [M + H]^+^ (calculated for C_21_H_21_O_4_, 337.1434). ^1^H NMR (700 MHz, methanol-*d*_*4*_) and ^13^C NMR (175 MHz, methanol-*d*_*4*_; see Table 1).

**TABLE 1:** ^1^H NMR and^13^C NMR data of compound **1** in MeOH (700 MHz).

NO.	H	C
1	—	126.4
2	7.53, d,*J* = 8.7 Hz	130.6
3	6.76, d,*J* = 8.7 Hz	115.6
4	—	163.0
5	6.76, d,*J* = 8.7 Hz	115.6
6	7.53, d,*J* = 8.7 Hz	130.6
1′	—	114.2
2′	—	163.9
3′	—	113.1
4′	—	160.4
5′	6.54, d,*J* = 8.9 Hz	102.0
6′	7.94, d,*J* = 8.9 Hz	130.2
*α*	7.72, d,*J* = 15.3 Hz	116.9
*β*	7.55, d,*J* = 15.3 Hz	144.6
1″	2.76 m,2.85 m	24.0
2″	4.51 t, *J* = 7.3 Hz	87.8
3″	—	144.7
4″	4.57 m,4.67 t, *J* = 1.8 Hz	112.1
4′-OCH_3_	3.80 s	54.9
3″-CH_3_	1.71 s	15.7
C=O	—	192.8

Compound **2**: yellow solid; UV (MeOH) λmax (logε) 346 (2.6) nm; ${\left[\alpha \right]}_{D}^{25.0}\;–$ 124.48 (c 0.01, MeOH); HRESIMS m/z = 385.1646 [M + H]^+^ (calcd for C_22_H_25_O_4_, 385.1646). ^1^H NMR (700 MHz, methanol-*d*_*4*_) and ^13^C NMR (175 MHz, methanol-*d*_*4*_); see Table 2.

**TABLE 2:** ^1^H NMR and^13^C NMR data of compound **2** in MeOH (700 MHz).

NO.	H	C
1	—	126.6
2	7.45, d, *J* = 8.6 Hz	130.2
3	6.72, d, *J* = 8.6 Hz	116.5
4	—	162.3
5	6.72, d, *J* = 8.6 Hz	116.5
6	7.45, d, *J* = 8.6 Hz	130.2
1′	—	115.5
2′	—	162.4
3′	—	114.7
4′	—	161.9
5′	6.62, d, *J* = 8.8 Hz	104.1
6′	7.85, d, *J* = 8.8 Hz	133.8
α	7.74, d, *J* = 15.5 Hz	124.8
β	7.61, d = 15.5 Hz	143.5
1″	5.07, d, *J* = 2.7 Hz	79.0
2″	4.45, d, *J* = 2.7 Hz	95.5
3″	—	70.2
4′-OCH_3_	3.87 s	55.3
1″-OCH_3_	3.36 s	55.4
3″-CH_3_	1.26 s	22.3
3″-CH_3_	1.12 s	23.4
C=O	—	187.8

Compound **3**: yellow solid; UV (MeOH) λmax (logε) 364 (2.5) nm; HRESIMS m/z = 423.2161 [M + H]^+^ (calcd for C_26_H_31_O_5_, 423.2166). ^1^H NMR (700 MHz, methanol-*d*_*4*_) and ^13^C NMR (175 MHz, methanol-*d*_*4*_); see Table 3.

**TABLE 3:** ^1^H NMR and^13^C NMR data of compound **3** in MeOH (700 MHz).

NO.	H	C
1	—	126.4
2	7.66, d, *J* = 8.7 Hz	130.5
3	6.87, d, *J* = 8.7 Hz	115.6
4	—	160.4
5	6.87, d, *J* = 8.7 Hz	115.6
6	7.66, d, *J* = 8.7 Hz	130.5
1′	—	114.4
2′	—	162.4
3′	—	116.6
4′	—	163.4
5′	6.68, d, *J* = 9.1 Hz	102.1
6′	8.05, d, *J* = 9.1 Hz	129.6
1″	3.39, d, *J* = 6.7 Hz	21.1
2″	5.26, t, *J* = 6.7 Hz	123.0
3″	—	133.3
4″	2.69 m	42.4
5″	5.58 m	128.4
6″	5.59 m	135.3
7″	—	81.1
α	7.69, d, *J* = 15.3 Hz	117.0
β	7.84, d, *J* = 15.3 Hz	144.6
3″-CH_3_	1.78 s	14.9
7″-CH_3_	1.30 s	29.4
7″-CH_3_	1.30 s	29.4
4′-OCH_3_	3.94 s	55.0
C=O	—	192.8

The other spectra of the three new compounds included ^1^H−^1^H COSY, HMBC, HMQC, NOESY, and HRESIMS (Supplementary Figures S1–S26).

**FIGURE 1:**
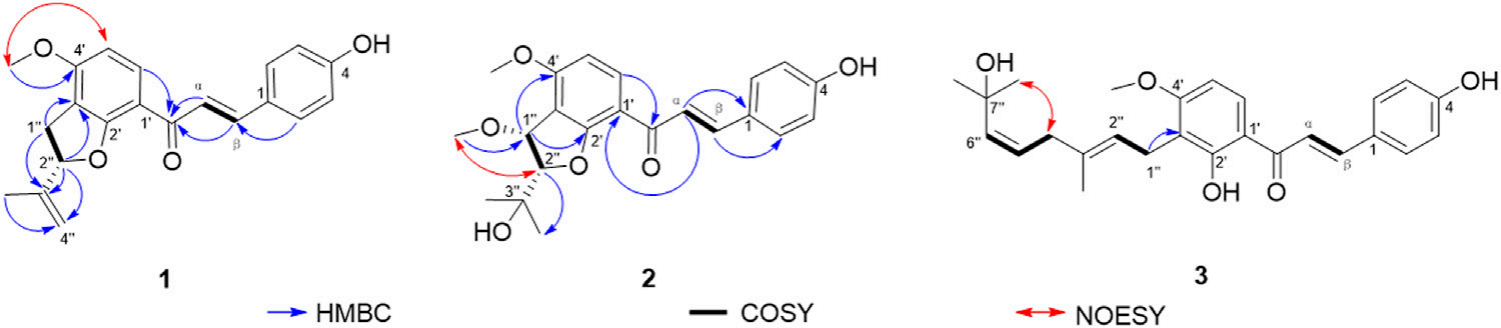
Main HMBC and ^1^H−^1^H COSY and NOESY correlations of compounds **1–3**.

**FIGURE 2:**
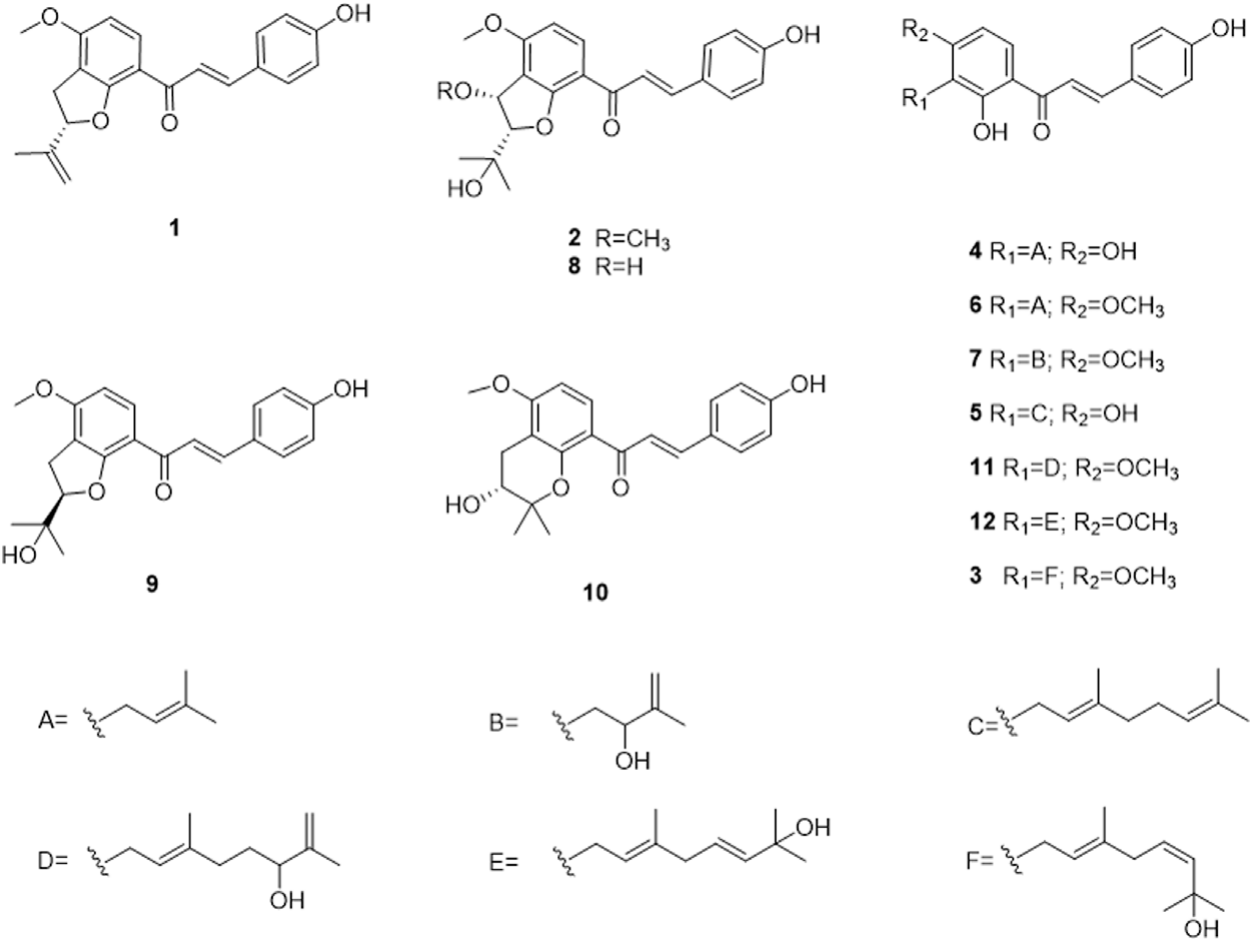
The chemical structures of compounds **1**–**12** isolated from seeds of *Abelmoschus manihot.*

Compound **1** was obtained as a yellow solid. The molecular formula of C_21_H_20_O_4_ was assigned on the basis of HRESIMS (m/z 337.1437 [M + H]^+^ calculated for 337.1434), ^13^C NMR, and HSQC spectra. A 1,2,3,4-tetrasubstituted benzene ring was interpreted by observing the ^1^H NMR spectrum of compound **1**, and signals [*δ*_H_ 7.94 (1H, d, *J* = 8.9 Hz, H-6′) and *δ*_H_ 6.54 (1H, d, *J* = 8.8 Hz, H-5′)] were obtained. In addition, signals in the aromatic region [*δ*_H_ 7.72 (2H, d, *J* = 8.7 Hz, H-2, H-6), *δ*_H_ 6.76 (2H, d, *J* = 8.7 Hz, H-3, H-5)] suggested that compound **1** contains an AABB coupling system benzene ring. Furthermore, two olefinic protons [*δ*_H_ 7.72 (1H, d, *J* = 15.3 Hz, H-*α*), *δ*_H_ 7.55 (1H, d, *J* = 15.3 Hz, H-*β*)] were observed. The large coupling constants (nearly 16.0 Hz) suggest the *trans* form of the double bonds. The ^13^C NMR and HSQC spectra revealed that these substances contain 21 carbon atoms, including two methyls (one methoxy), two methylenes (one sp^2^ and one sp^3^), nine methines (one sp^3^ and eight sp^2^), and six quaternary carbons (sp^2^ olefinic, including three oxygenated). The above NMR data prompted us to conclude that compound **1** was a chalcone derivative. We carefully analyzed the NMR spectra and found they were very similar to those of xanthoangelol D (Baba et al., 1990). The only difference was that the chemical shift of C-2″ in compound **1** was *δ*_C_ 87.8 ppm, whereas that in xanthoangelol D was *δ*_C_ 75.0 ppm. The structure of **1** was confirmed by carefully interpreting its 2D NMR data. In the ^1^H−^1^H COSY spectrum (Figure 1), the correlation of H-1′′/H-2″ was observed, which confirmed the connection sequence of C-1″ and C-2′′. The HMBC correlations of H-1′′/C-3″, and H-2′′/C-3″, C-4″, and C-5″ and the aforementioned ^1^H−^1^H COSY correlation indicated the presence of a C5 fragment, which was attached to C-3′. The HMBC correlation from the –OCH_3_ to C-4″ indicated the OCH_3_ was connected to C-4′. This result was further confirmed by the NOESY correlation from –OCH_3_ to H-5' (NOESY data). Although the correlation of H-2′′/C-2′ was not observed, the chemical shift of C-2″ and the lack of a degree of unsaturation confirmed the existence of the C5 ring. A chemical shift similar to that of C-2″ was 88.1 ppm in lespecyrtin B_1_, which resembled compound **1** (Mori-Hongo et al., 2009). Thus, the structure of **1** was assigned and named abelmanihotol A. The ^1^H and ^13^C NMR data for compound **1** are presented in Table 1, and its structure is shown in Figure 2.

Compound **2** was obtained as a yellow solid. Its molecular formula was C_22_H_24_O_6_ (10 degrees of unsaturation) according to HRESIMS (m/z 385.1646 [M + H]^+^ calculated for 385.1646) , ^13^C NMR, and HSQC spectra. The ^13^C NMR and HSQC spectra revealed four methyls, ten methines, and eight quaternary carbons in this substance. The signals resembled those of jejuchalcone E (Shimomura et al., 2013). The only difference was that an additional methoxy group was connected to C-1′′. This alteration was confirmed by the HMBC correlations of –OCH_3_/C-1′′. Notably, **2** had two chiral centers (Figure 1), and their relative configurations were confirmed by the coupling constant between H-1″ and H-2′′ (*J*_H-1″,H-2″_). The *J*_H-1″, H-2″_ coupling constant of **2** (*J* = 2.7 Hz) was closer to that of *trans* (*J* = 3.6 Hz) than to *cis* (*J* = 5.5 Hz) (Shimomura et al., 2013), supporting the *trans* configurations of H-1″ and H-2′′. This conclusion was further confirmed by the NOESY correlations of H-2′′/1″-OCH_3_. Thus, the structure of **2** was assigned and named abelmanihotol B. The ^1^H and ^13^C NMR data for compound **2** are presented in Table 2, and its structure is shown in Figure 2.

Compound **3** was obtained as a yellow solid. Its molecular formula was identified as C_26_H_30_O_5_ (12 degrees of unsaturation) on the basis of HRESIMS (m/z 423.2161 [M + H]^+^ calculated for 423.2166). The ^1^H NMR spectrum of **3** displayed six aromatic proton signals (*δ*_H_ 7.45 [2H, d, *J* = 8.7 Hz, H-2, H-6], *δ*_H_ 6.87 [2H, d, *J* = 8.7 Hz, H-3, H-5], *δ*_H_ 8.05 [1H, d, *J* = 9.1 Hz, H-6′], *δ*_H_ 6.68 [1H, d, *J* = 9.1 Hz, H-5′]), suggesting that the structure contained a 1,4-disubstituted benzene ring and a 1,2,3,4-tetrasubstituted benzene ring. Two olefinic protons (*δ*_H_ 7.69 [1H, d, *J* = 15.3 Hz, H-*α*], *δ*_H_ 7.84 [1H, d, *J* = 15.3 Hz, H-*β]*]) were observed. The large coupling constants (nearly 16.0 Hz) of the olefinic proton suggested the *trans* form of the double bonds. The NMR data of **3** were similar to those of (1-[2-hydroxy-3-(7-hydrox-3,7-dimethyl-2,5-ocadienyl)-4-methoxyphenyl]-3-(4-hydroxyphenyl)-2-propen-l-one chalcone (Ryu and Kweon, 2018). The only difference was that double bond *Δ* ^5''(6″)^ in compound **3** was a *cis*, whereas (1-[2-hydroxy-3-(7-hydrox-3,7-dimethyl-2,5-ocadienyl)-4-methoxyphenyl]-3-(4-hydroxyphenyl)-2-propen-l-one chalcone was *trans*. This conclusion was confirmed by the NOESY correlations of H-4′′/7″-CH_3_ (Figure 1). Therefore, the structure of **3** was assigned and named abelmanihotol C. The ^1^H and ^13^C NMR data for compound **3** are presented in Table 3, and its structure is shown in Figure 2.

### Compound **1** Inhibits NO and IL-1β Release in THP-1 Cells

**FIGURE 3:**
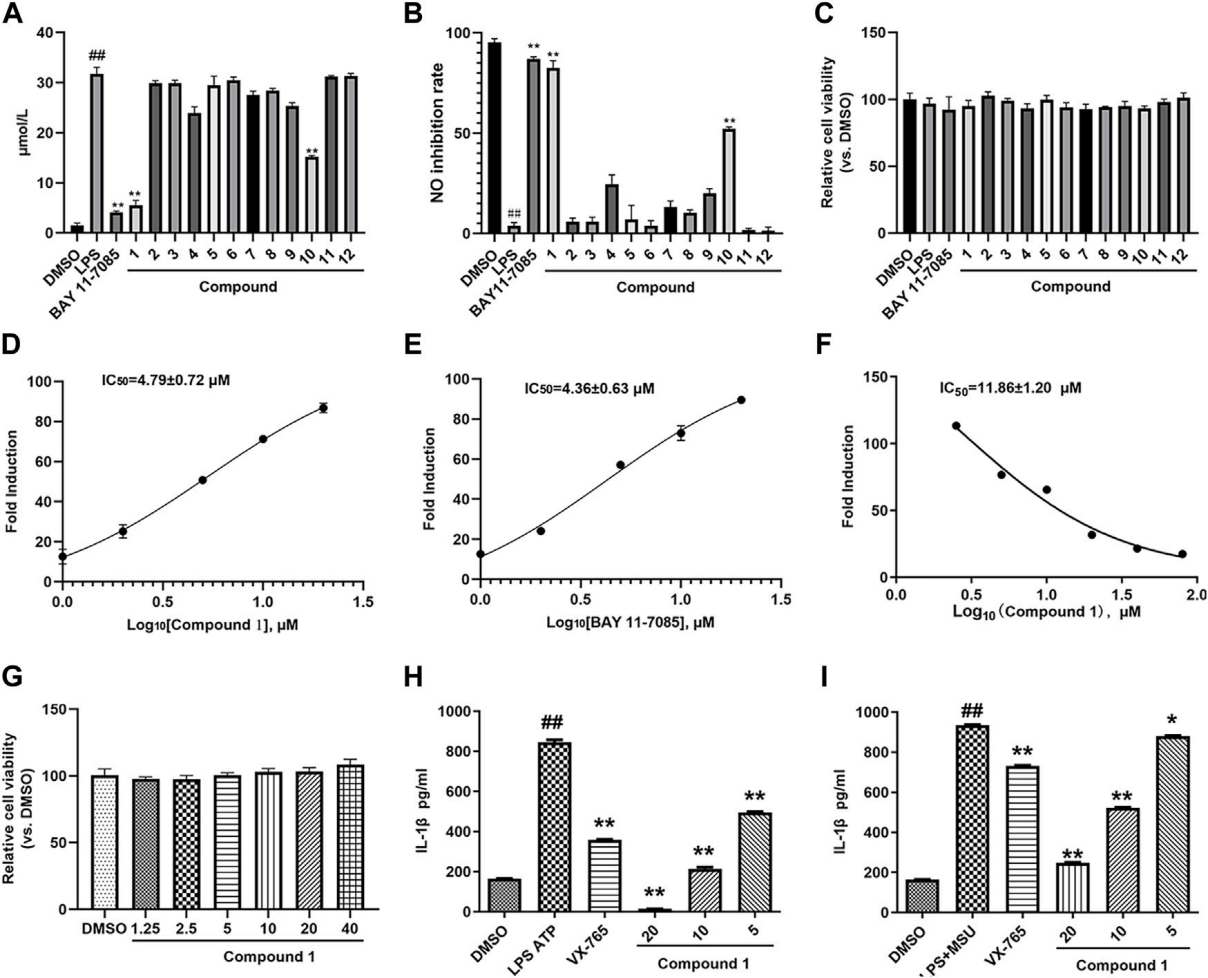
Compound **1** inhibited IL-1β release. **(A)** PMA-differentiated THP-1 cells were treated with compounds **1**–**12** (10 μM) and BAY 11-7085 (10 μM) and stimulated with LPS (0.5 μg/ml) for 24 h, cell supernatants were collected for NO detection. **(B)** NO production inhibition rate. **(C)** The cytotoxicity of compounds **1**–**12** (10 μM) and BAY 11-7085 (10 μM) in THP-1 cells. **(D,E)** The IC_50_ value of compound **1 (D)** and BAY 11-7085 **(E)** on NO production. **(F)** The IC_50_ value of compound **1** on IL-1β release. **(G)** The cytotoxicity of different doses of compounds **1** in THP-1 cells. **(H,I)** LPS (1 μg/ml) priming PMA-differentiated THP-1 cells were treated with various doses of compound **1** and VX-765 (500 nM) and stimulated with MSU (150 mg/ml) or ATP (5 mM), cell supernatants were collected for IL-1β production. Cont., DMSO control. ^##^ *p* < 0.01 compared with the control group, * *p* < 0.05, **p < 0.01 compared with the model group.

SAM is used as a Tibetan medicine for treating skin diseases closely related to inflammation. We first detected inhibitory activity against NO in compounds **1–12** extracted from SAM. As shown in Figures 3A,B, compound **1** and **10** significantly inhibited NO production, but the inhibitory rate of compound **1** is higher than that of compound **10**, so compound **1** was selected for further study. Figure 3C reveals that no compounds had any effect on THP-1 cell viability, which indicates that the inhibitory effect of compound **1** on NO release was not due to an influence on THP-1 cell viability. We then further detected the IC_50_ of compound **1** on LPS-induced NO production. BAY 11-7085 was used as the positive control. The IC_50_ value of compound **1** and BAY 11-7085 were 4.79 ± 0.72 and 4.36 ± 0.63 μM, respectively (Figures 3D,E). NLRP3 inflammasome activation causes pro-caspase-1 self-cleavage, which induces the transformation of IL-1β from immature to mature. Hence, IL-1β production is an essential signal for NLRP3 inflammasome activation. We further examined the inhibitory effects of compound **1** on NLRP3 inflammasome activation in PMA-differentiated and LPS-primed THP-1 cells. As shown in Figures 3F,G, compound **1** significantly restrained IL-1β release in a concentration-dependent manner, had an IC_50_ value of 11.86 ± 1.20 μM, and did not affect THP-1 cell viability. In addition, the effect of compound **1** on IL-1β production was primed by other classic NLRP3 inflammasome stimulators, such as MUS and ATP. As expected, compound **1** suppressed IL-1β release induced by MUS or ATP in LPS-primed and PMA-differentiated THP-1 cells, and compound **1** showed a higher inhibition rate than VX765 when the dose was more than 10 μM (Figures 3H,I). These results confirmed that compound **1** is a broad-spectrum NLRP3 inflammasome inhibitor.

### Compound **1** Suppresses Caspase-1 Activation in THP-1 Cells

**FIGURE 4:**
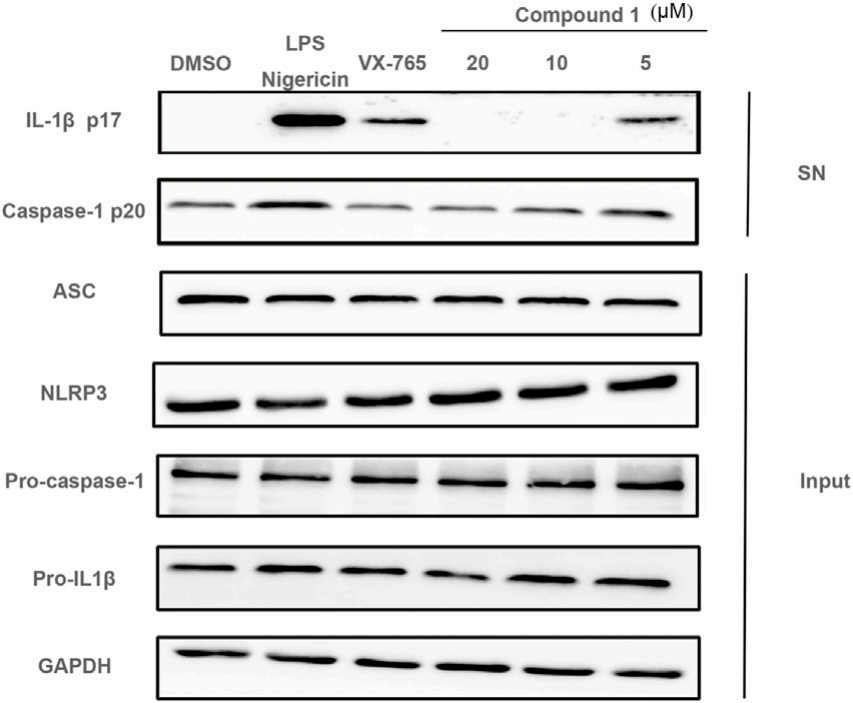
LPS (1 μg/ml) priming PMA-differentiated THP-1 cells were treated with various doses of compounds **1** and VX-765 (500 nM) and stimulated with nigericin (5 μM), cell culture supernatants (SN) and cell lysate (Input) were collected. NLRP3 inflammasome related proteins were detected using western blot.

The inhibitory effects of compound **1** on IL-1β secretion were confirmed using western blot, which detected the release of IL-1β in the supernatant. As shown in Figure 4, compound **1** treatment attenuated IL-1β secretion in the cell supernatant in a dose-dependent manner. Western blot results further revealed that compound **1** treatment restrained nigericin-induced caspase-1 activation without affecting NLRP3, ASC, and pro-caspase-1 in the cell lysates. These results indicated that compound **1** inhibited IL-1β secretion by affecting NLRP3 inflammasome assembly.

### Compound **1** Restrains ASC Oligomerization

**FIGURE 5:**
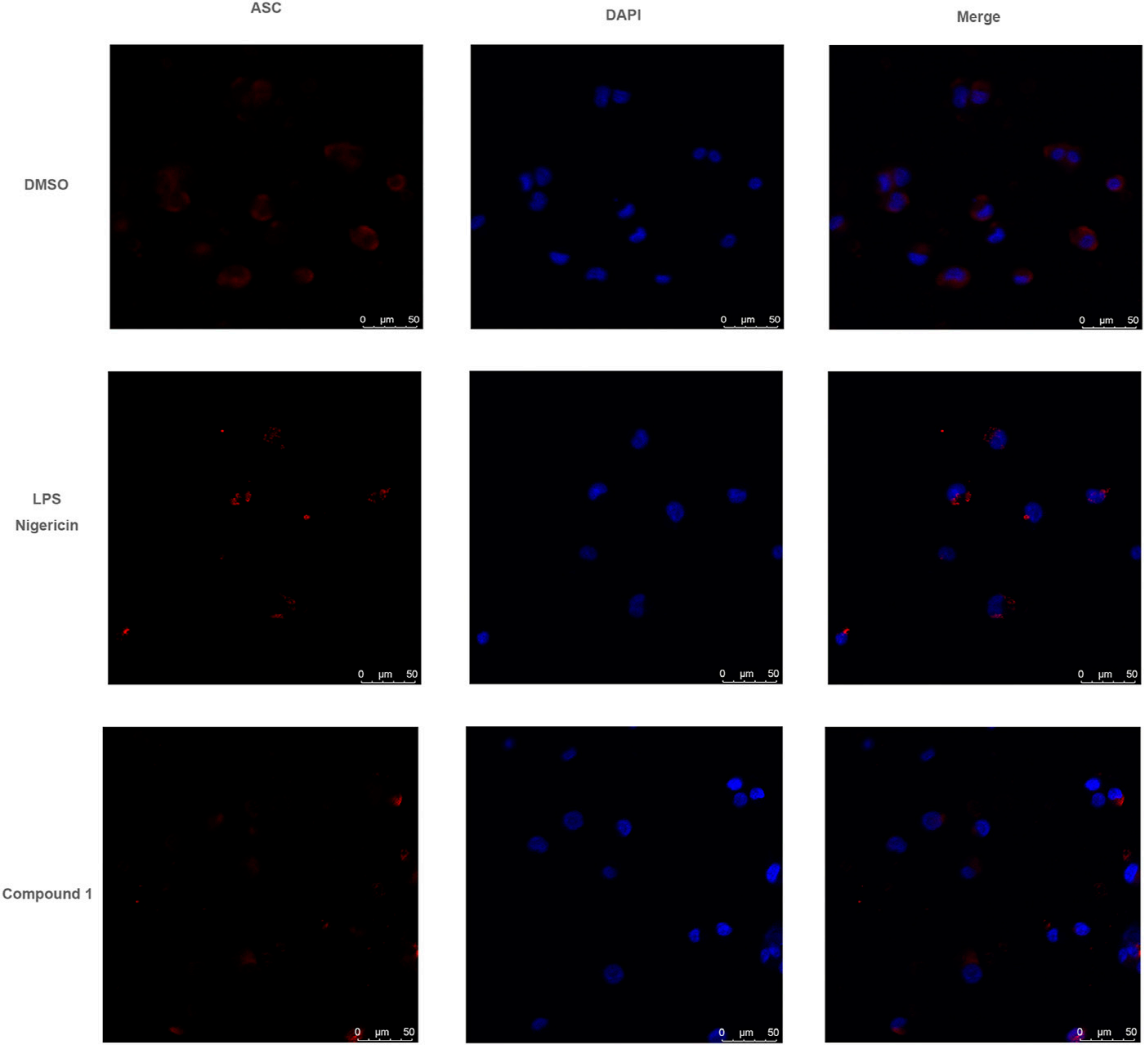
Compound 1 restrained ASC oligomerization. LPS (1 μg/ml) priming PMA-differentiated THP-1 cells were treated with compound **1** (10 μM) or DMSO and stimulated with nigericin, confocal immunofluorescent images of ASC, DAPI are displayed.

ASC oligomerization is a key step in NLRP3 inflammasome installation and results in pro-caspase-1 self-cleavage and mature IL-1β release. We determined whether compound **1** affected the inflammasome assembly by inhibiting ASC oligomerization. The effect of compound **1** on ASC oligomerization was detected through immunofluorescence. Figure 5 reveals that compound **1** treatment evidently attenuated nigericin-induced ASC speck formation. Immunofluorescence experiment indicates the inhibitory effects of compound **1** on caspase-1 activation and mature IL-1β secretion through the blocking of ASC oligomerization.

## Discussion

Mammalian immunity is facilitated by innate and adaptive immune systems. The response of this immune system is regulated by PAMPs. Pattern recognition receptors (PRRs) can recognize not only the invariant structural sequences of invading pathogens (microbial nucleic acid and microbial cell wall components), but also endogenous danger signals released by host injury or stress (MSU, ATP, and high-mobility group box 1; Lamkanfi and Dixit (2014). PRPs serve as important sensors that can detect PAMPs and DAMPs and trigger various signaling pathways and their downstream effectors (Janeway and Medzhitov, 2002). Inflammasomes are intracellular multiprotein signal transduction platforms and can cause inflammation in response to pathogen infection and cell damage. An essential part of innate immunity, inflammasomes are polyprotein complexes composed of intracellular pattern recognition receptors, which mediate the immune response of the body to microbial infection and cell damage, and maintain the body’s immune homeostasis (Chen et al., 2009). Among the currently identified inflammatory factors, the NLRP3 inflammasome is the most widely and deeply studied. The NLRP3 inflammasome is composed of the receptor NLRP3, adaptor protein ASC, and pro-caspase-1. An activated NLRP3 inflammasome triggers the self-cleavage of pro-caspase-1 and produces an active effect on protein caspase-1. Caspase-1 mediates IL-1β maturation and secretion, and gasdermin D cleavage causes pyroptosis (Kim and Jo, 2013). The persistent activation of NLRP3 promotes the overexpression of inflammatory factors, leading to diabetes, atherosclerosis, gout, and tumor occurrence (Fusco et al., 2020). Therefore, NLRP3 inflammasome inhibitors are important in the treatment of NLRP3-related diseases (Yang et al., 2019; Zahid et al., 2019; Ma et al., 2020).

In this study, three new chalcone derivatives, abelmanihotols A−C (compounds **1–3**), and nine known compounds (compounds **4**–**12**): isobavachalcone (compound **4**), xanthoangelol (compound **5**), 4-hydroxyderricin (compound **6**), xanthoangelol D (compound **7**), jejuchalcone E (compound **8**), 1-[2,3-dihydro-2-(1-hydroxy-1-methylethyl)-4-methoxy-7-benzofuranyl]-3-(4-hydroxyphenyl)-2-propen-1-one (compound **9**), 4′-methoxy-bavachromanol (compound **10**), xanthoangelol G (compound **11**), and (1-[2-hydroxy-3-(7-hydrox-3,7-dimethyl-2,5-ocadienyl)-4-methoxyphenyl]-3-(4-hydroxyphenyl)-2-propen-l-one chalcone (compound **12**) were isolated from SAM, a traditional Tibetan medicine documented in *Jing Zhu Ben Cao* and usually used for treatment of atopic dermatitis, rheumatism, and leprosy (Silva et al., 2018; Zheng et al., 2021; Kong et al., 2022). The occurrence of these diseases is closely related to the abnormal activation of inflammatory factors. NO is an effector of inflammatory responses and serves as a key regulator of immune response (Ramachandran et al., 2018). Hence, we first determined the effects of compounds **1–12** on NO secretion in THP-1 cells. Compound **1** markedly inhibited NO production, with an IC_50_ value of 4.79 ± 0.72 μM. BAY 11-7085, which was used as the positive control, inhibited NO production and had an IC_50_ value of 4.36 ± 0.63 μM. This result indicated that compound **1** is an important substance in SAM and exerts therapeutic effects. Furthermore, we observed that the release of IL-1β was obviously attenuated after compound **1** treatment in different NLRP3 inflammasome stimulation models. This result confirmed that compound **1** is a broad-spectrum NLRP3 inflammasome inhibitor. In addition, western blot indicated that compound **1** treatment restrained nigericin-induced caspase-1 activation without influencing NLRP3, ASC, and pro-caspase-1 expression. As ASC oligomerization is an important step for NLRP3 inflammasome installation, we examined the ASC oligomerization after compound **1** treatment (Green et al., 2018; Soriano-Teruel et al., 2021). Immunofluorescence showed that compound **1** treatment evidently attenuates nigericin-induced ASC speck formation. Overall, these results indicate that compound **1** can be developed as a potent and efficacious NLRP3 inflammasome inhibitor and is a promising compound for treating NLRP3-related diseases.

## Conclusion

Three new chalcone derivatives, abelmanihotols A−C (**1–3**), and nine known compounds (**4–12**) were isolated and identified from the 85% ethanol extract of SAM. Compound **1** and **10** significantly inhibited NO production in PMA-differentiated and LPS-primed THP-1 cells. The inhibition rate of compound **1** for NO release was close to the positive control (BAY 11-7085). In addition, compound **1** treatment obviously attenuated IL-1β secretion in different NLRP3 inflammasome stimulation models. Western blot analysis and immunofluorescence results further revealed the inhibitory effects of compound **1** on pro-caspase-1 self-cleavage and IL-1β release were exerted through the down-regulation of pro-ASC speck formation. Therefore, SAM and its bioactive compounds can play an important role in the treatment of NLRP3-related diseases.

## Data Availability

The datasets presented in this study can be found in online repositories. The names of the repository/repositories and accession number(s) can be found in the article/Supplementary Material.
