# Supplementary material for: Chalcone Derivatives From Abelmoschus manihot Seeds Restrain NLRP3 Inflammasome Assembly by Inhibiting ASC Oligomerization
Source: Front Pharmacol. 2022 Jul 7;13:932198. doi: 10.3389/fphar.2022.932198 (PMC9301202; doi:10.3389/fphar.2022.932198)
Supplement: Supplementary file 1 [file Table1.DOCX]

# Supplementary Material

| **Figure S1.** ^1^H NMR spectrum of **1** in methanol-*d_4_* |
| --- |
| **Figure S2.** ^13^C NMR spectra of **1** in methanol-*d_4_* |
| **Figure S3.** HSQC spectrum of **1** in methanol-*d*_4_ |
| **Figure S4.** HMBC spectrum of **1** in methanol-*d_4_* |
| **Figure S5.** ^1^H-^1^H COSY spectrum of **1** in methanol-*d_4_* |
| **Figure S6.** NOESY spectrum of **1** in methanol-*d_4_* |
| **Figure S7*.*** HRESIMS of **1** |
| **Figure S8*.*** Optical rotatory data of **1** |
| **Figure S9.** UV spectrum of **1** |
| **Figure S10.** ^1^H NMR spectrum of **2** in methanol-*d_4_* |
| **Figure S11.** ^13^C NMR spectra of **2** in methanol-*d_4_* |
| **Figure S12.** HSQC spectrum of **2** in methanol-*d_4_* |
| **Figure S13.** HMBC spectrum of **2** in methanol-*d_4_* |
| **Figure S14.** ^1^H-^1^H COSY spectrum of **2** in methanol-*d_4_* |
| **Figure S15.** NOESY spectrum of **2** in methanol-*d_4_* |
| **Figure S16.** HRESIMS of **2** |
| **Figure S17*.*** Optical rotatory data of **2** |
| **Figure S18.** UV spectrum of **2** |
| **Figure S19.** ^1^H NMR spectrum of **3** in methanol-*d_4_* |
| **Figure S20.** ^13^C NMR spectra of **3** in methanol-*d_4_* |
| **Figure S21.** HSQC spectrum of **3** in methanol-*d_4_* |
| **Figure S22.** HMBC spectrum of **3** in methanol-*d_4_* |
| **Figure S23.** ^1^H-^1^H COSY spectrum of **3** in methanol-*d_4_* |
| **Figure S24.** NOESY spectrum of **3** in methanol-*d_4_* |
| **Figure S25.** HRESIMS of **3** |
| **Figure S26.** UV spectrum of **3** |
| **Figure S27.** The chromatogram of Fr. 1 |
| **Figure S28.** The chromatogram of Fr. 2 |
| **Figure S29.** The chromatogram of Fr. 3 |


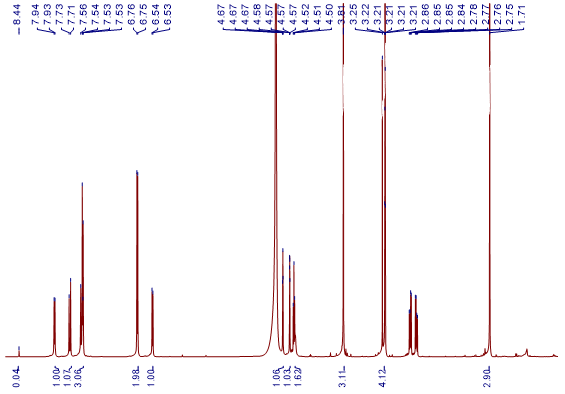


**Figure S1.** ^1^H NMR spectrum of **1** in methanol-*d_4_*


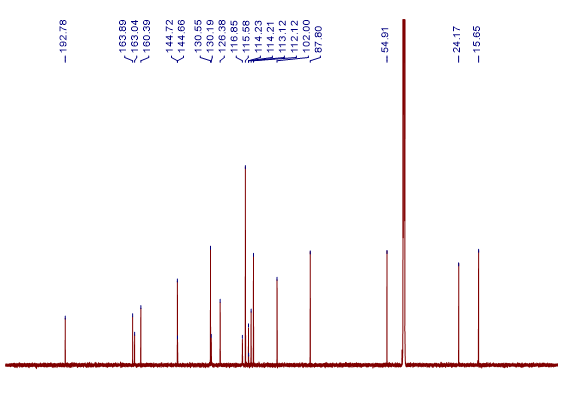


**Figure S2.** ^13^C NMR spectra of **1** in methanol-*d_4_*


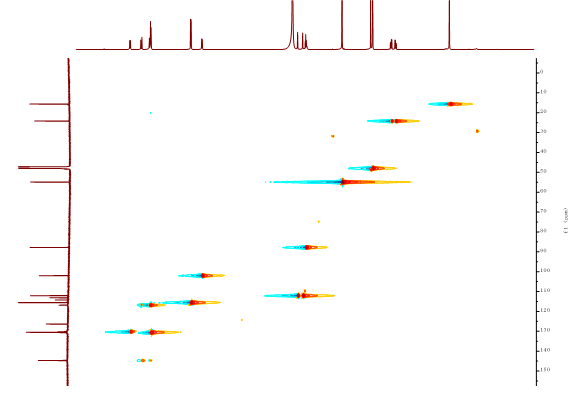


**Figure S3.** HSQC spectrum of **1** in methanol-*d*_4_


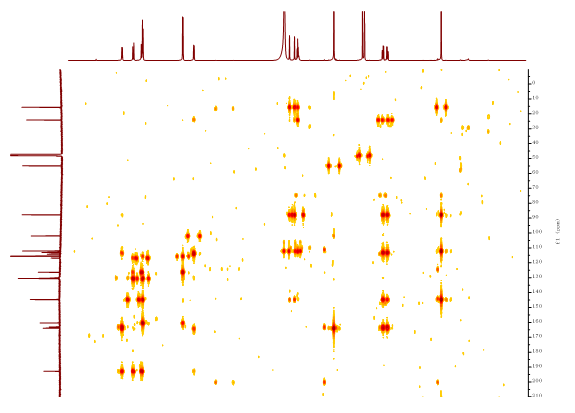


**Figure S4.** HMBC spectrum of **1** in methanol-*d_4_*


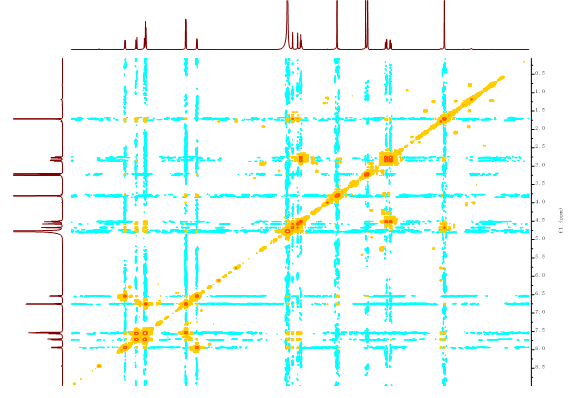


**Figure S5.** ^1^H-^1^H COSY spectrum of **1** in methanol-*d_4_*


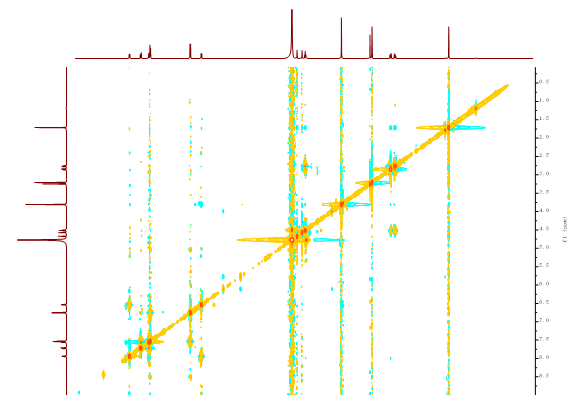


**Figure S6.** NOESY spectrum of **1** in methanol-*d_4_*


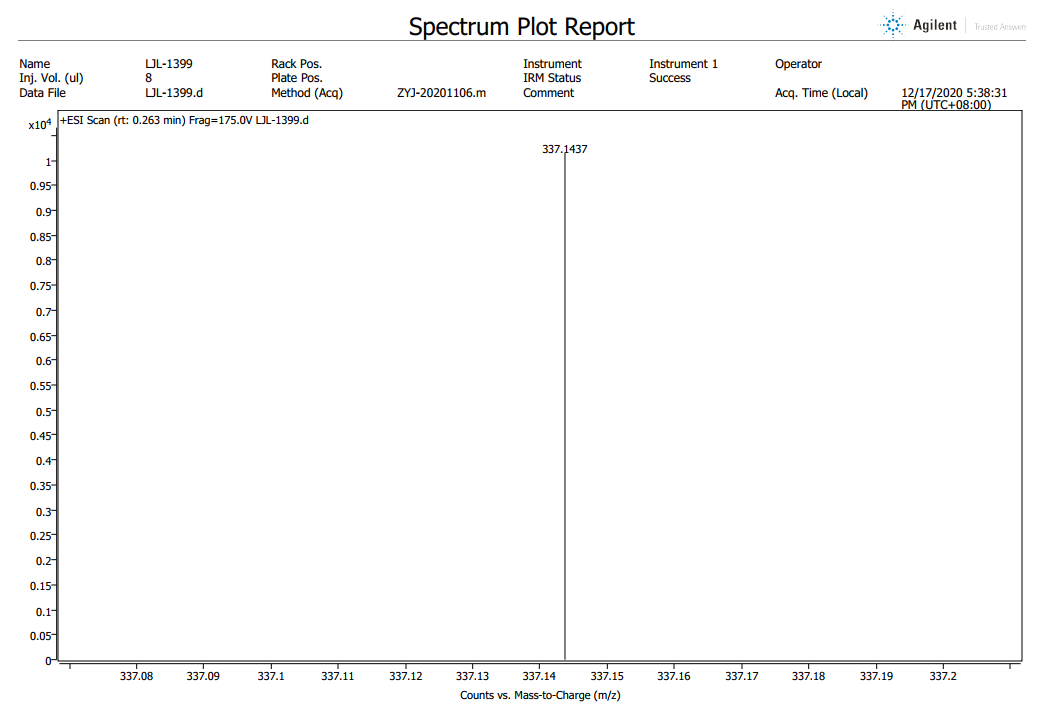


**Figure S7*.*** HRESIMS of **1**


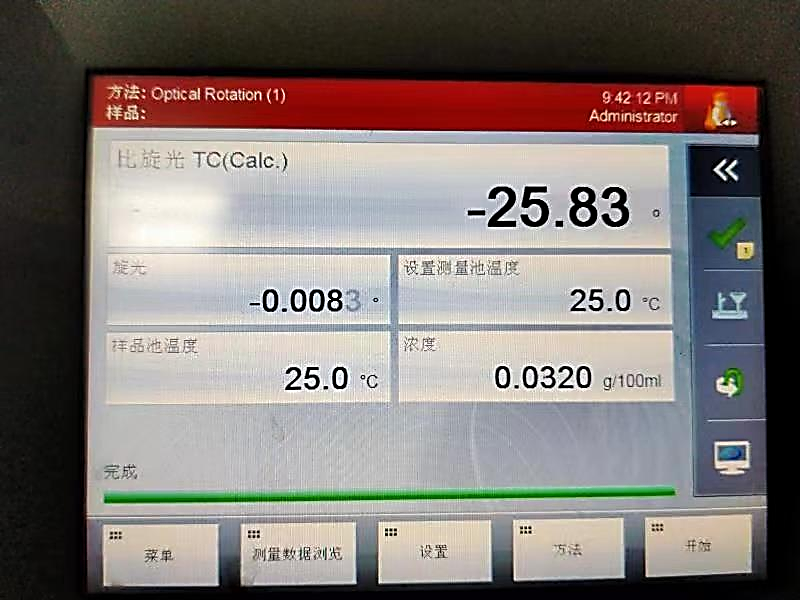


**Figure S8*.*** Optical rotatory data of **1**


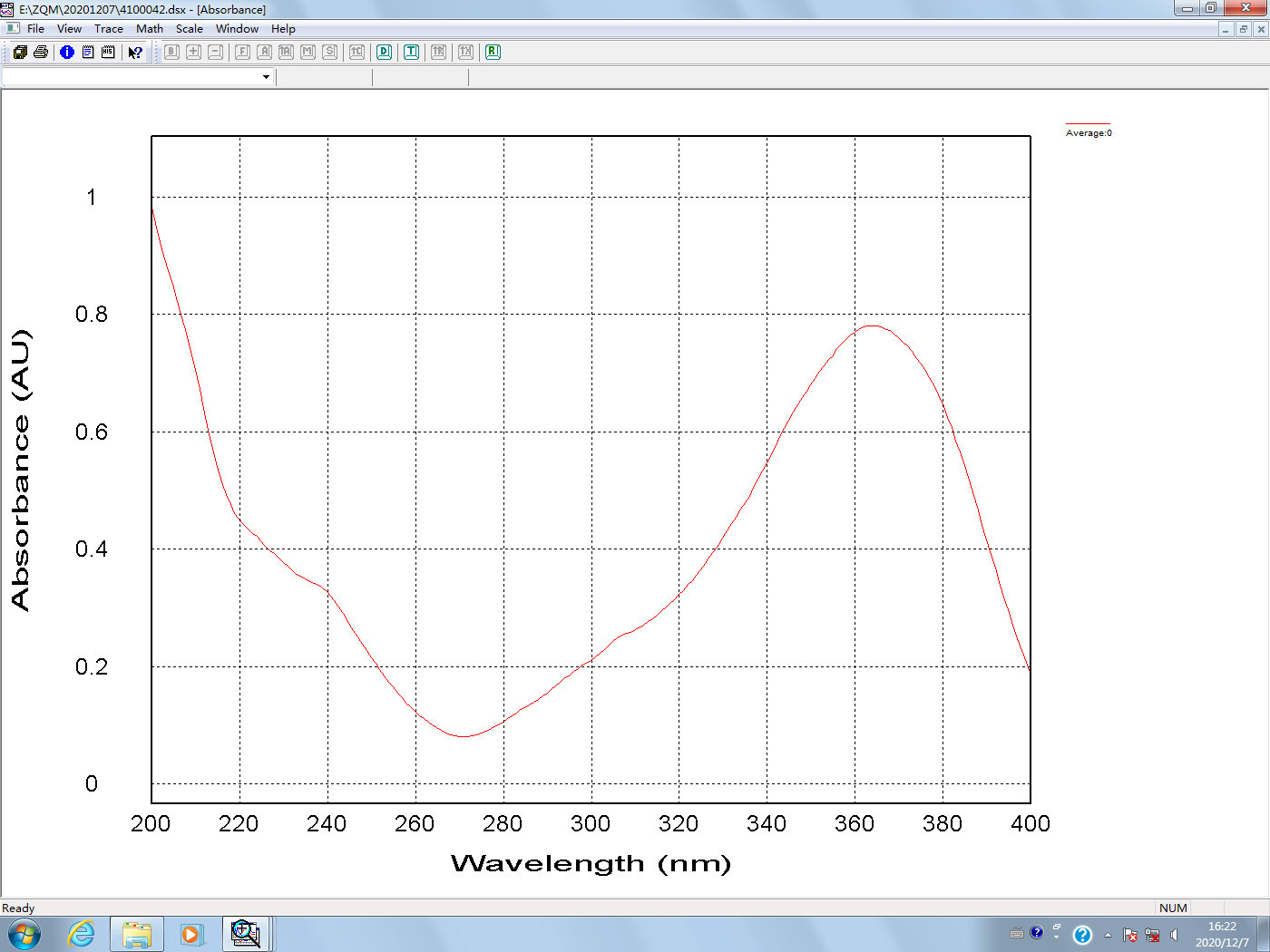


**Figure S9.** UV spectrum of **1**


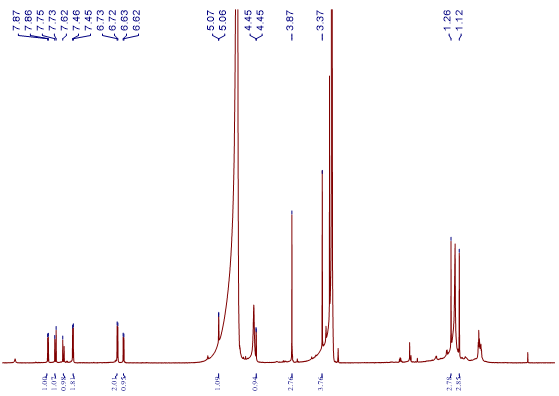


**Figure S10.** ^1^H NMR spectrum of **2** in methanol-*d_4_*


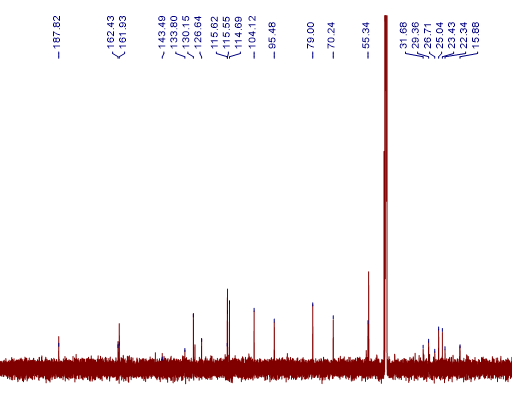


**Figure S11.** ^13^C NMR spectra of **2** in methanol-*d_4_*


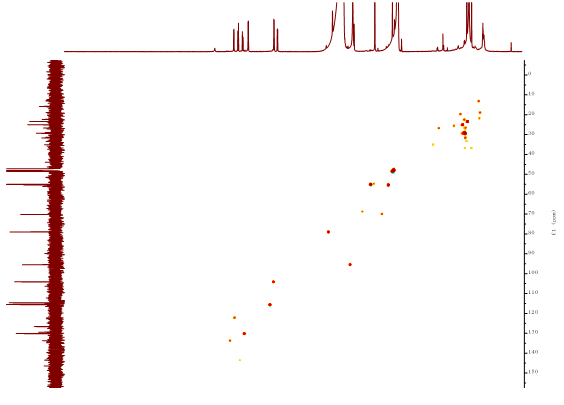


**Figure S12.** HSQC spectrum of **2** in methanol-*d_4_*


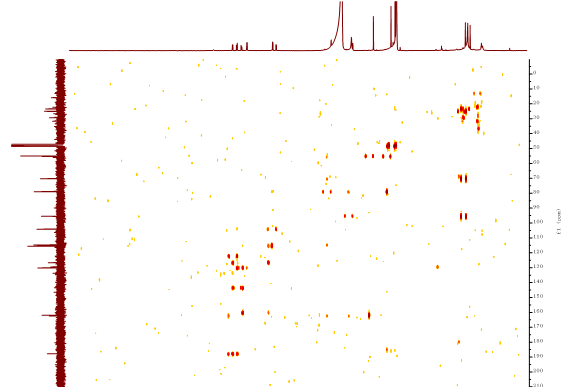


**Figure S13.** HMBC spectrum of **2** in methanol-*d_4_*


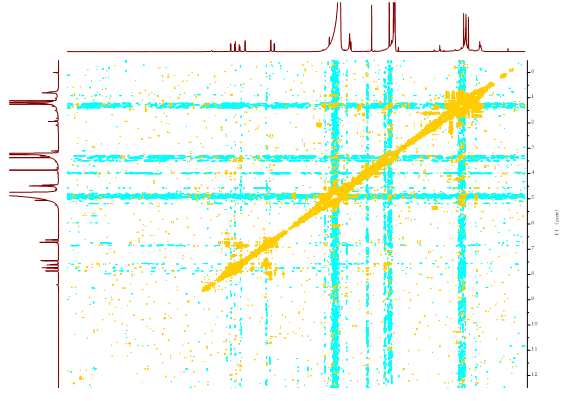


**Figure S14.** ^1^H-^1^H COSY spectrum of **2** in methanol-*d_4_*


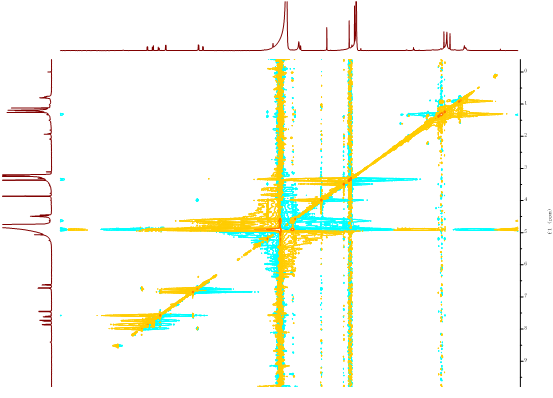


**Figure S15.** NOESY spectrum of **2** in methanol-*d_4_*


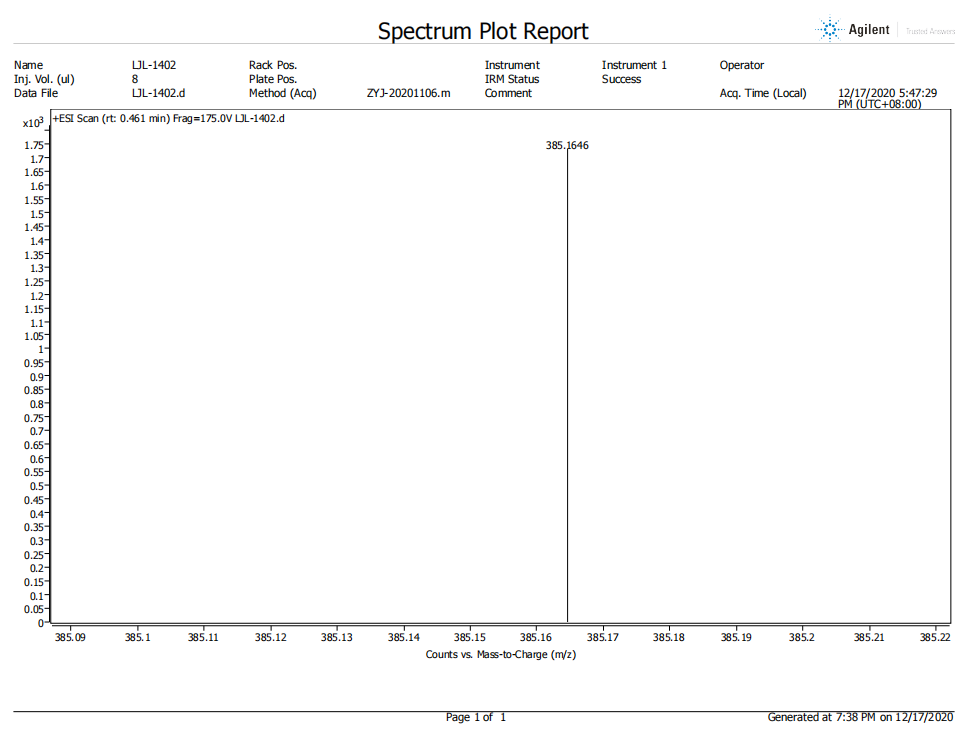


**Figure S16.** HRESIMS of **2**

**
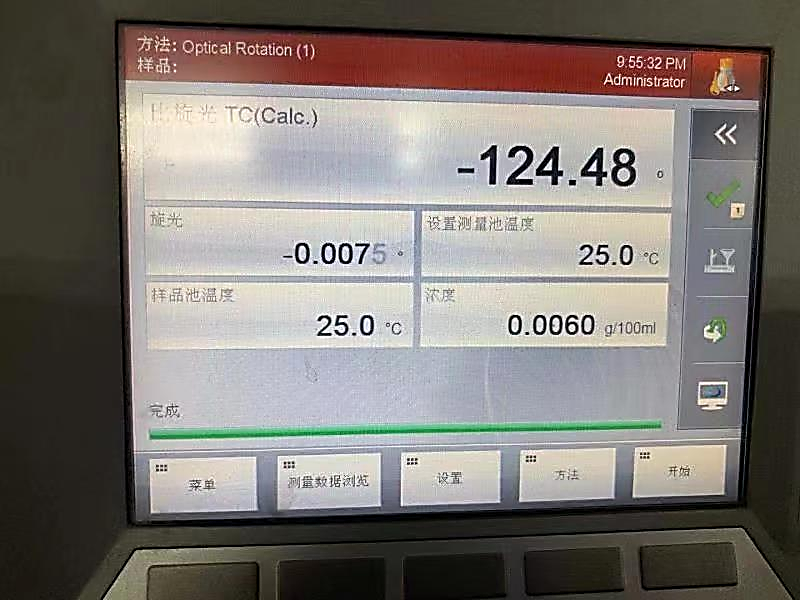
**

**Figure S17*.*** Optical rotatory data of **2**


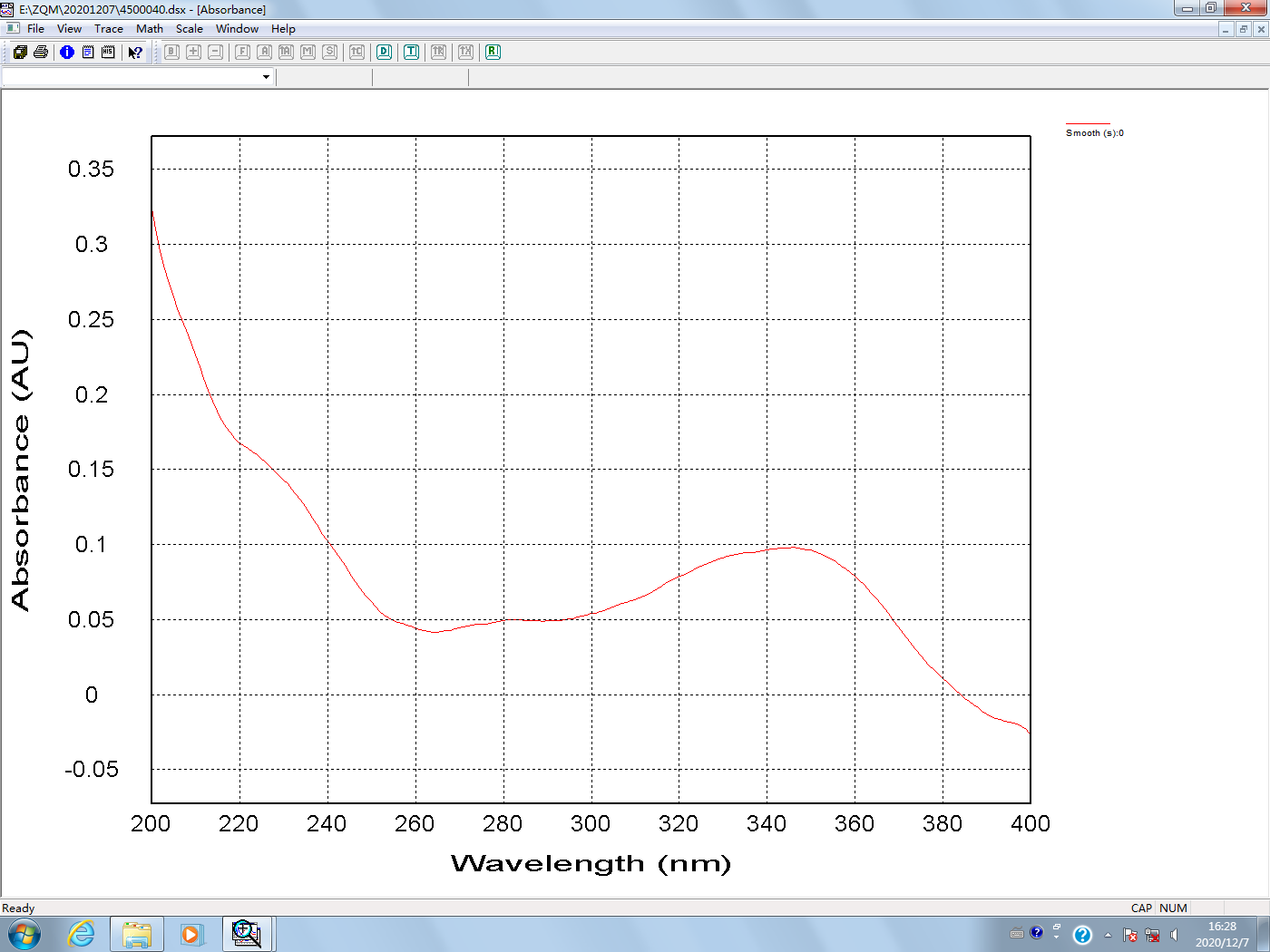


**Figure S18.** UV spectrum of **2**


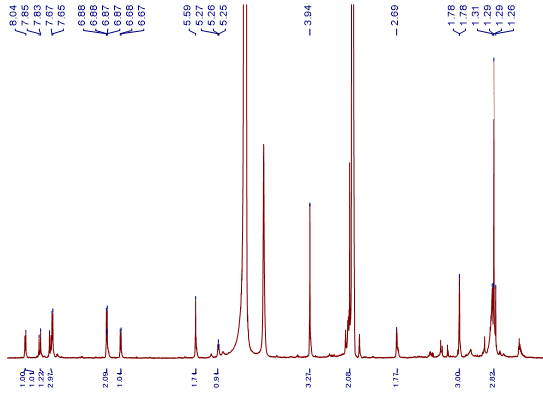


**Figure S19.** ^1^H NMR spectrum of **3** in methanol-*d_4_*


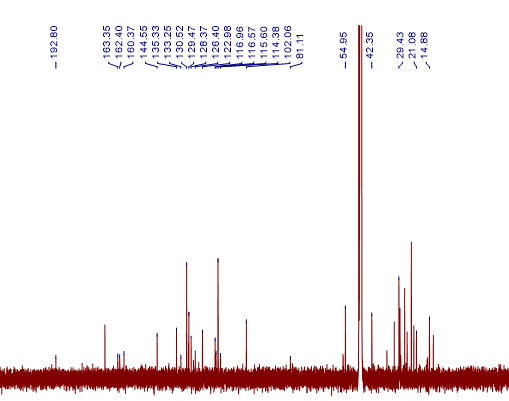


**Figure S20.** ^13^C NMR spectra of **3** in methanol-*d_4_*


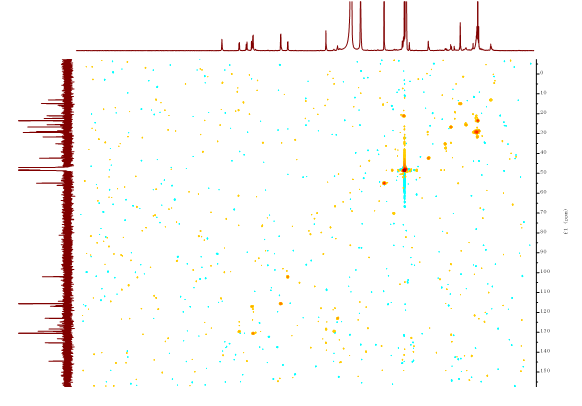


**Figure S21.** HSQC spectrum of **3** in methanol-*d_4_*


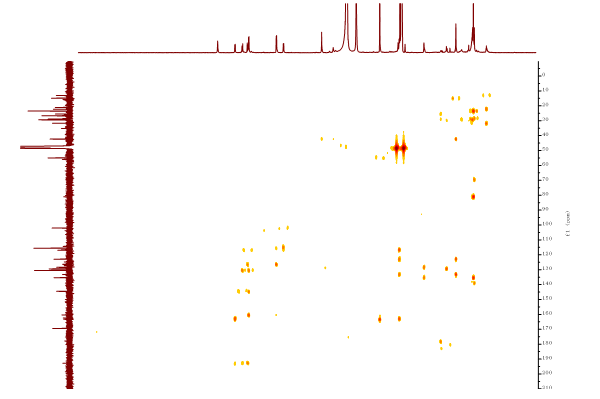


**Figure S22.** HMBC spectrum of **3** in methanol-*d_4_*


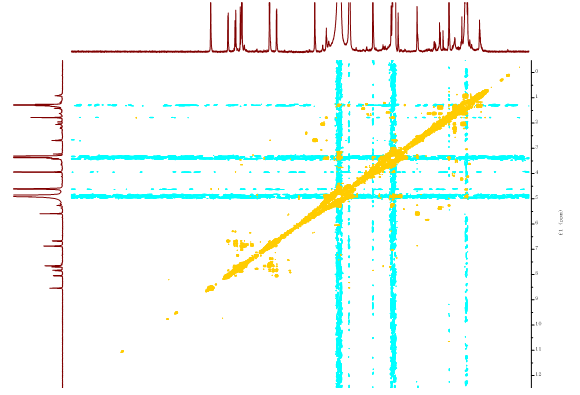


**Figure S23.** ^1^H-^1^H COSY spectrum of **3** in methanol-*d_4_*


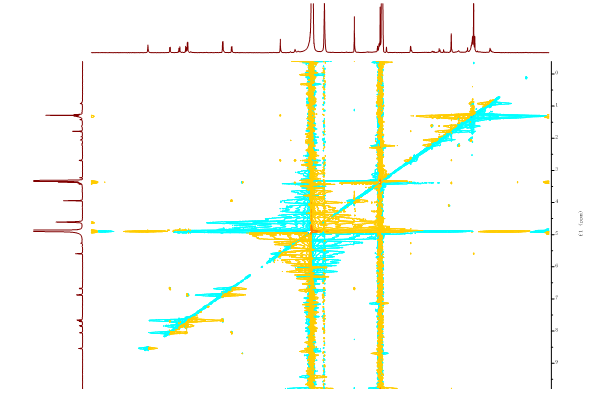


**Figure S24.** NOESY spectrum of **3** in methanol-*d_4_*


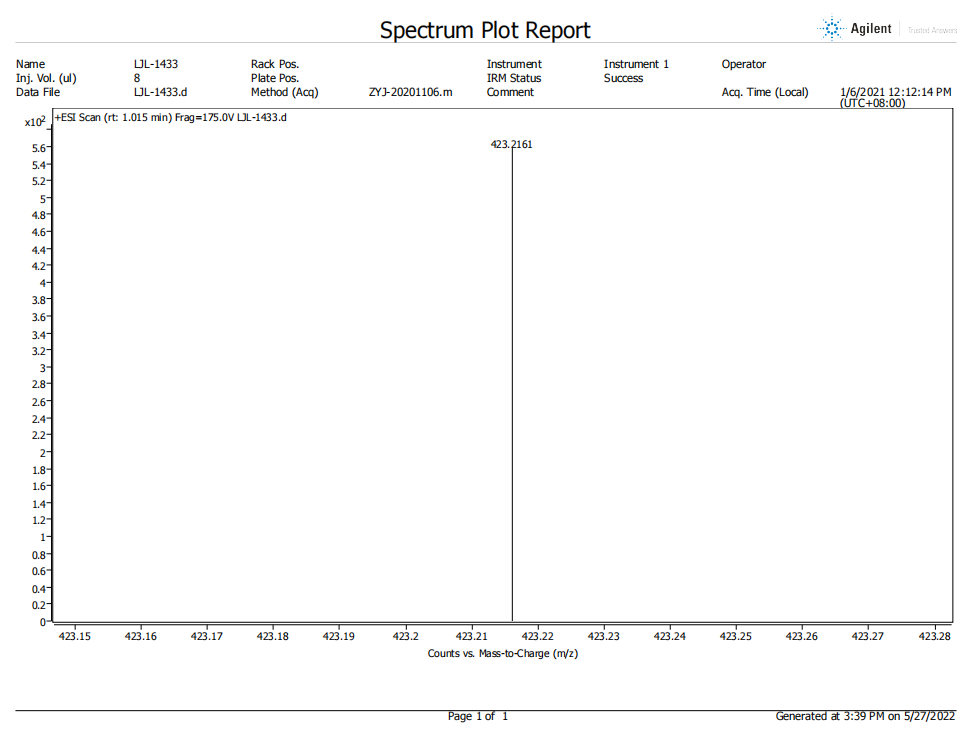


**Figure S25.** HRESIMS of **3**


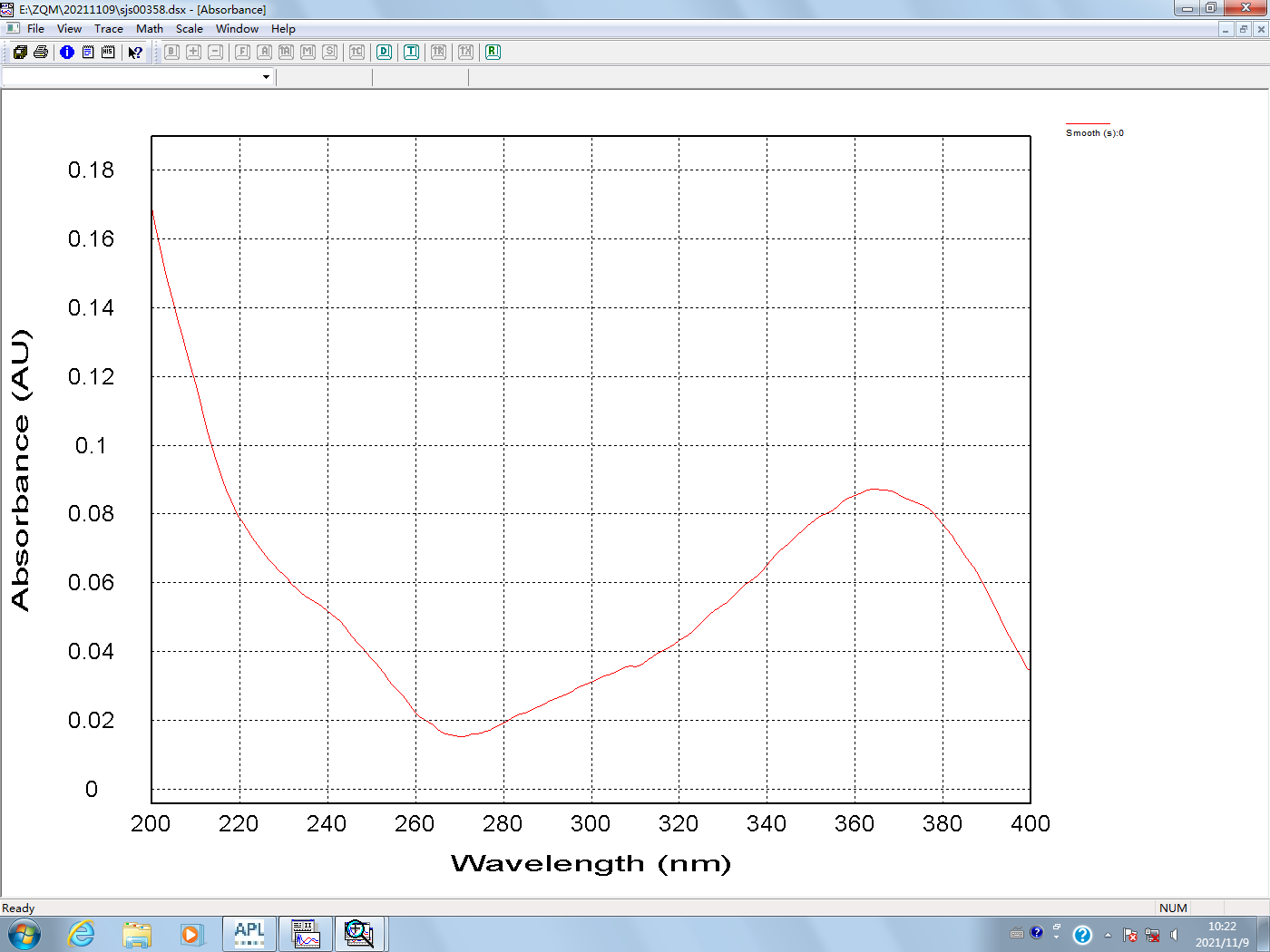


**Figure S26.** UV spectrum of **3**


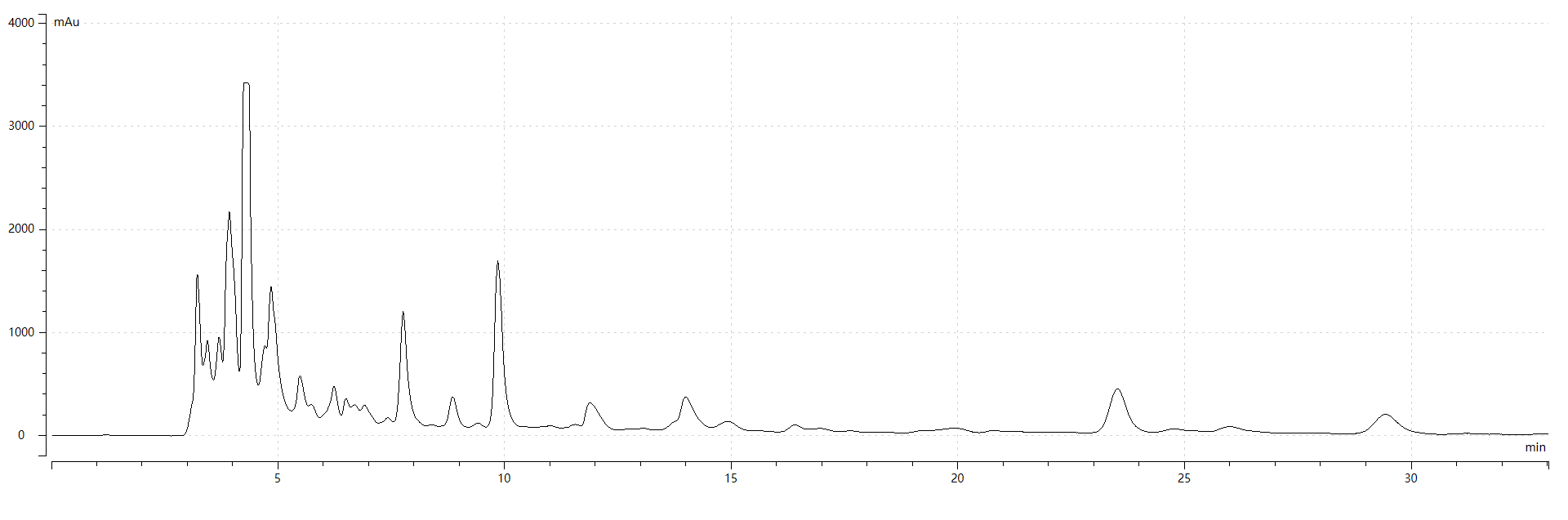


**Figure S27.** The chromatogram of Fr. 1 (30%, MeOH/H_2_O, flow rate: 3 mL/min)


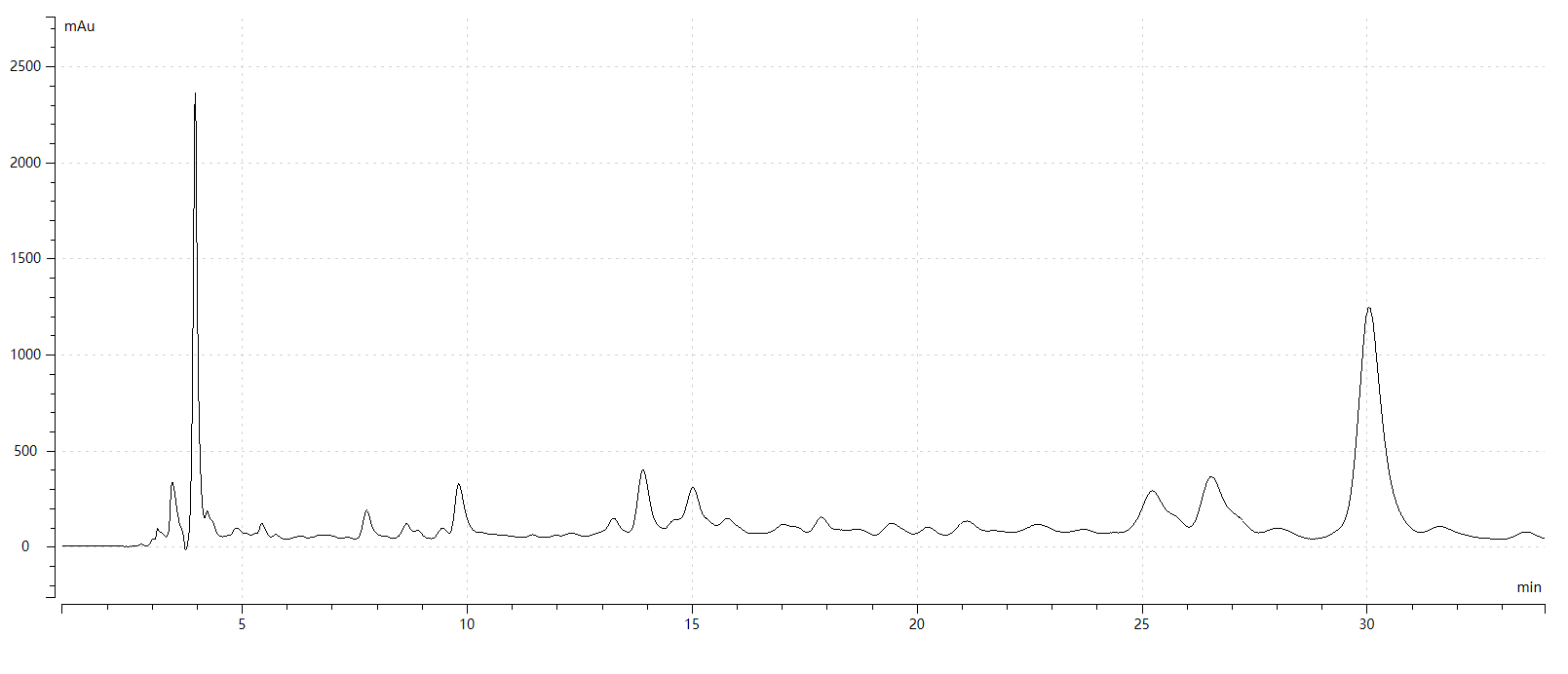


**Figure S28.** The chromatogram of Fr. 2 (30%, MeOH/H_2_O, flow rate: 3 mL/min)


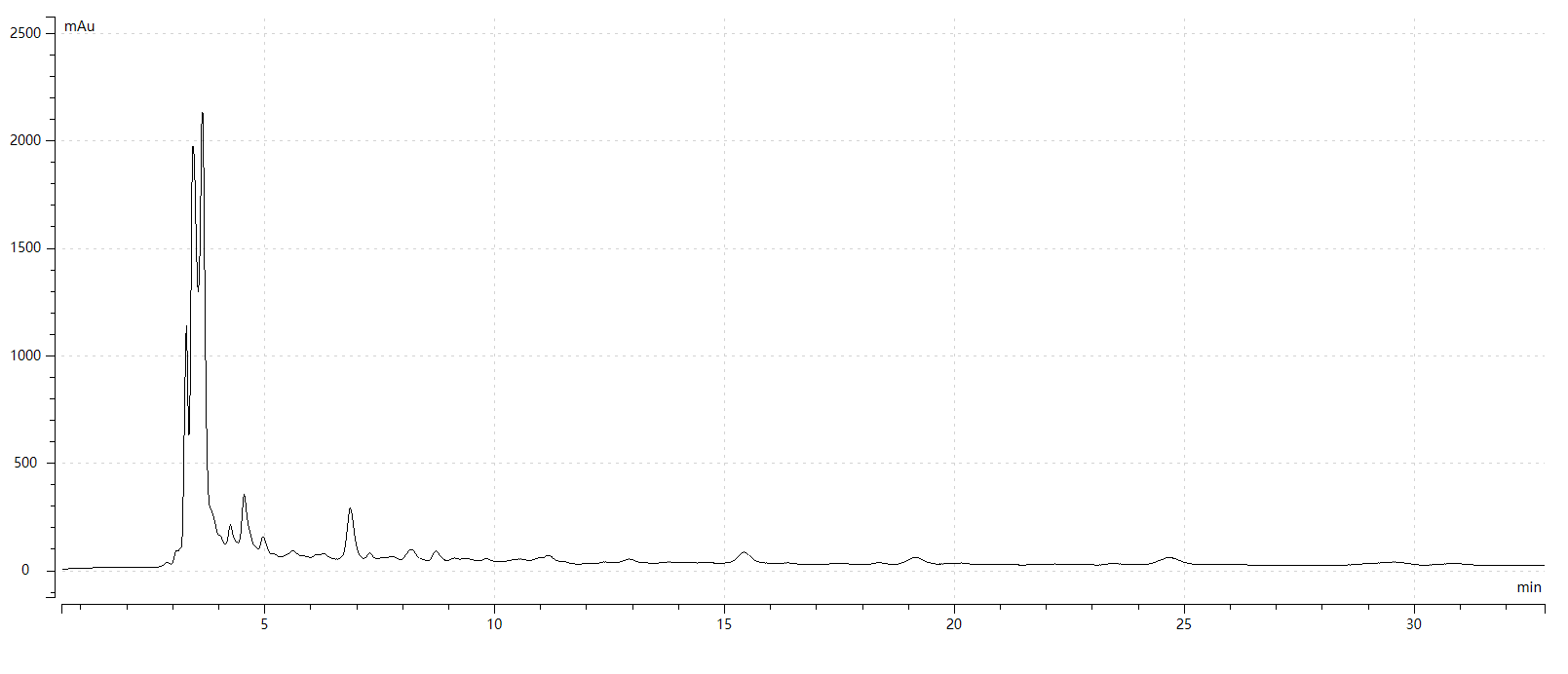


**Figure S29.** The chromatogram of Fr. 3 (30%, MeOH/H_2_O, flow rate: 3 mL/min)
